# Rebuttal to the Formal Comment on Schorr et al. (2014) submitted by Tyack et al. (2015)

**DOI:** 10.1371/journal.pone.0142437

**Published:** 2015-12-17

**Authors:** Gregory S. Schorr, Erin A. Falcone, David J. Moretti, Russel D. Andrews

**Affiliations:** 1 Cascadia Research Collective, Olympia, Washington, 98501, United States of America; 2 Naval Undersea Warfare Center, Newport, Rhode Island, 02841, United States of America; 3 School of Fisheries and Ocean Sciences, University of Alaska Fairbanks, Fairbanks, Alaska, 99709, United States of America; 4 Alaska SeaLife Center, Seward, Alaska, 99664, United States of America; Texas A&M University-Corpus Christi, UNITED STATES

Tyack *et al*. cite three specific concerns in their commentary regarding Schorr *et al*. [1], and we respond briefly to them here. Quotes around normal font designate text from their commentary; italicized text in quotes is from the original paper [1].

Tyack *et al*. state that readers may misinterpret findings in Schorr *et al*. [1] because they aren’t “…adequately appreciating that that some of the extreme dives highlighted in the paper were likely response dives.” In Schorr *et al*. [1], we stated repeatedly that whales were tagged on, and many remained within, one of the most heavily used Mid-Frequency Active (MFA) sonar training ranges in the world, and were “*almost certainly exposed at some point…”* to Navy sonar. An explicit goal of this descriptive paper was to “…*provide insight into the true behavioral range of this species in a region of regular acoustic disturbance*”, and we concluded with the statement "*Given that whales tagged in this study far exceeded diving behavior previously described as extreme*[2], *the role humans might play in shaping this behavior can’t be discounted*." Our use of the word ‘true’ was meant to indicate the range of behaviors we now understand this species to be capable of. The word ‘true’ was not meant suggest that behaviors described were ‘undisturbed’. While we regret any misunderstanding this may have caused, we did not anticipate this interpretation of ‘true’ because of our very explicit statements that our data were collected in an area of frequent acoustic disturbance. We did not go further than a broad description of these behaviors and their general context because we had no control over the complex sound field that these whales were living in, and comprehensive records of Navy sonar use were unavailable, as we explained in our concluding paragraph.

We continue to work toward a comparison of behavior in exposed and unexposed states using these data. Acquisition of the sonar use data required for this comparison and development of appropriate analytical methods has been a lengthy process and we chose not to delay the dissemination of these generalized behavioral data in their entirety, given the unique perspective long-term behavioral records provide for this species. We clearly and repeatedly stated that human disturbance was likely to have influenced the behavior we recorded, and continue to feel strongly that selectively releasing the sonar context of a small subset of our data outside a manuscript that details the analytical techniques, limitations, and assumptions associated with deriving that information would be irresponsible. Even as Tyack *et al*. assert that we should have released specifics about sonar events in the original paper and call on us to do so here, they also go on to state: “…we suggest that conclusions about effects of sonar on diving behavior in the Schorr *et al*. [1] dataset should wait until the authors complete their analysis of the “*subset of this dataset where major sources of acoustic disturbance—or just as importantly*, *lack thereof—can be accurately documented and independently verified*””. We could not agree more, and this is why we omitted specific details about known periods of sonar use during these tag deployments, acknowledged that sonar was used an unknown number of times, and attempted to provide balanced discussion of overall trends in our data relative to what has been published about responses to simulated sonar in this species previously.

Tyack *et al*. also worry that readers may interpret our findings in inappropriate ways because they don’t understand “the ways in which the longer-term, lower resolution tags used by Schorr *et al*. (2014) complement rather than replace the utility of controlled exposure experiments using shorter-term high-resolution archival tags”. We do not question the utility of shorter-term, high-resolution archival tags and agree completely that the use of longer-term lower resolution tags is a complementary approach. The value and relevance of high-resolution, multi-sensor tag data is well-supported in the literature, some of which we referenced in [1]. We limited our comparison with short-term, high-resolution datasets to summarized parameters that are derived from those tags at the same resolution our tags collect data (e.g. dive durations, inter-deep-dive-intervals), but felt such a comparison was warranted given the dramatic increase in available data from this species (6,827 dives versus the 327 reference dives included in DeRuiter *et al*. [3]). We remain steadfast in our belief that researchers should interpret limited samples of behaviors from low numbers of individuals with caution, particularly in cases where behavior may vary regionally, and vary considerably more than previously thought within and among individual whales even in the same region. This does not diminish the value of high-resolution, short-term tags to capture fine-scale responses in controlled exposure experiments on this species, but underscores the value of also using extended duration tags to place these responses in the broader behavioral context in which they occur, particularly when conclusions are based on summarized parameters [3,4], and especially when using short-term results to infer long-term consequences.

Tyack *et al*. also wish to underscore “…that these recent findings do not call into question the response dives documented in DeRuiter *et al*. (2013)”. We did not question whether the whales in DeRuiter *et al*. [3] responded to simulated sonar. We, in fact, emphasized the significance of the foraging disruption that occurred during these controlled exposure experiments relative to the extensive sample of dive interval data in Schorr *et al*. [1]. Tyack *et al*. go on to suggest that we should not have made any reference to the reactive dive durations from DeRuiter *et al*. [3], as the extremes dives in our dataset may have occurred in the presence of MFA sonar. That may be true of any dive in our data, and this is why we compared the reactive dive durations of [3] to the median dive durations of our whales, while noting that our whales also conducted many significantly longer dives. Our intent was not to contradict the DeRuiter *et al*. [3] conclusion that whales responded to simulated MFA sonar exposure, only to note that the durations of the response dives were not extreme when compared with the larger dive duration dataset we obtained from whales in the same area. If any readers may have interpreted the discussion of findings from Schorr *et al*. [1] otherwise, that was certainly not our intent. Our goal was simply to summarize the general diving behavior across the entire dataset with the clear acknowledgement that it contained an unknown number of potential anthropogenic disturbances. This is likely to be the case with long-term telemetry data from any free-ranging species living in the midst of human activities, and should not preclude the utility of these data for descriptive studies nor the discussion of potential anthropogenic effects within them.
